# Why do dogs wag their tails?

**DOI:** 10.1098/rsbl.2023.0407

**Published:** 2024-01-17

**Authors:** Silvia Leonetti, Giulia Cimarelli, Taylor A. Hersh, Andrea Ravignani

**Affiliations:** ^1^ Comparative Bioacoustics Group, Max Planck Institute for Psycholinguistics, Nijmegen, The Netherlands; ^2^ Department of Life Sciences and Systems Biology, University of Turin, Turin, Italy; ^3^ Department of Human Neurosciences, Sapienza University of Rome, Rome, Italy; ^4^ Domestication Lab, Konrad Lorenz Institute of Ethology, Department of Interdisciplinary Life Sciences, University of Veterinary Medicine Vienna, Vienna, Austria; ^5^ Marine Mammal Institute, Oregon State University, Newport, OR, USA; ^6^ Center for Music in the Brain, Department of Clinical Medicine, Aarhus University, Aarhus, Denmark

**Keywords:** domestication, dogs, canids, tail wagging, rhythm, evolution of communication

## Abstract

Tail wagging is a conspicuous behaviour in domestic dogs (*Canis familiaris*). Despite how much meaning humans attribute to this display, its quantitative description and evolutionary history are rarely studied. We summarize what is known about the mechanism, ontogeny, function and evolution of this behaviour. We suggest two hypotheses to explain its increased occurrence and frequency in dogs compared to other canids. During the domestication process, enhanced rhythmic tail wagging behaviour could have (i) arisen as a by-product of selection for other traits, such as docility and tameness, or (ii) been directly selected by humans, due to our proclivity for rhythmic stimuli. We invite testing of these hypotheses through neurobiological and ethological experiments, which will shed light on one of the most readily observed yet understudied animal behaviours. Targeted tail wagging research can be a window into both canine ethology and the evolutionary history of characteristic human traits, such as our ability to perceive and produce rhythmic behaviours.

## 1. Introduction

Domestic dogs (*Canis familiaris*; hereafter dogs) are the most widespread carnivore in the world: with an estimated population of one billion individuals, they are present in nearly all areas where humans occur [1,2]. Through the simple act of sharing physical space, humans directly interact with dogs in many contexts and must use different cues and modalities to effectively communicate [3,4]. Visual signals are used as communicative cues in both human–dog and dog–dog interactions [5,6]. In particular, tail attributes such as carriage (i.e. position) and wagging provide readily observable informational cues, which humans use to infer the inner states of dogs [7–9]. Tail wagging, defined as the repetitive movement of the tail across the midsagittal/median plane, may well be one of the most conspicuous of all animal behaviours for humans to observe [9–12]. The human sensitivity to and intuition for dog tail movements (with tail wagging generally associated with positive valence) is so strong that engineers have leveraged it when designing user interfaces for utility and social robots [13–15]. Despite the ubiquity of dogs in our lives and all the meaning we ascribe to tail wagging, quantitative studies to date have led to patchy results and a structured theoretical framework is missing.

We summarize existing literature on dog tail wagging by considering the mechanistic, ontogenetic, functional and evolutionary aspects of this behaviour. We tackle the question of why dogs wag their tails more frequently and in more contexts than other closely related canids, such as wolves. This overview serves as a starting point to propose empirical low-hanging fruits, recommendations and suitable methodologies for future studies.

## 2. Tail wagging and Tinbergen's four questions

Why do dogs wag their tails? We can answer this question by considering tail wagging behaviour in terms of Tinbergen's four questions [16]: how does it work, mechanistically? How does it develop? What is it for? How did it evolve?

### (a) Mechanism

Dog tails are an extension of the spine, but little is known about how tail movements are neuro-physiologically controlled. The cerebellum is likely involved, given that electrical stimulation in the fastigial nucleus is accompanied by an increase in wagging [17]. Wagging is an asymmetric behaviour, with dogs showing side biases depending on the stimuli they encounter. This suggests brain lateralization in dogs. Dogs exhibit a right-side wagging bias, determined by left hemisphere activation, for stimuli that have a positive emotional valence (e.g. when shown their owner or a familiar person). On the contrary, they show left-biased wagging, hence right hemisphere activation, for stimuli that elicit withdrawal (e.g. when shown an unfamiliar, dominant dog or when in aggressive situations) [8,18–20]. Dogs also perceive wagging asymmetries in robot dogs [21] and conspecifics [22]. For example, dogs show more behavioural and physiological signs of stress when watching video silhouettes of left-biased wagging dogs compared to right-biased wagging dogs [22].

Several studies have documented positive correlations between time spent tail wagging and heart rate [17,23], although links between wagging and heart rate variability are less clear [12,23]. Tail wagging is frequently associated with both positive and negative arousal, suggesting a correlation with arousal-related hormones and neurotransmitters [24–32]. For example, there is indirect evidence linking oxytocin and tail wagging, especially when dogs are reunited with a familiar human [33,34]. However, associations between tail wagging behaviour and cortisol levels are inconsistent across studies [24,35–40]. This is likely because baseline cortisol levels can co-vary with many other parameters (e.g. sex, breed, age and life history of a dog) [38,41–44]. Alternatively, or in addition, past inconsistencies may have arisen because tail wagging is typically analysed as one broad behavioural category, without taking into account its multidimensional nature and parameters (which might be modulated by different arousal, and hence cortisol, levels). This could explain why one study found that aggressive dogs wagged their tails more (and had lower serotonin levels) than non-aggressive dogs—a result that is counterintuitive to the widely held human belief linking tail wagging to positive valence in dogs [19].

### (b) Ontogeny

To our knowledge, no study has tracked the development of tail wagging behaviour in the same individual(s) throughout life. One study, however, quantified several behavioural features of dog and wolf pups, including tail wagging, during object-preference tasks [45]. Pups of both species were hand-raised and then tested on their preference for their human carer versus other stimuli at three, four and five weeks of age. Four- to five-week-old dog pups frequently started to wag their tails and began displaying preferences for their carer. By contrast, wolf pups almost never wagged their tails. These results align with a short-term study (less than one week) that investigated how adult beagles interact with a human: wagging shifted from left- to right-side biased as dogs became more familiar with an experimenter [18].

### (c) Function

Both tail movement and tail carriage convey information in dog–dog [46,47], dog–human [18,38,40,48] and dog–object [49] interactions. Across canids, tail wagging with low carriage is often used as a visual sign of appeasement, submission or non-aggressive intent [50,51]. The combination of tail wagging and tail carriage seems a reliable status indicator of formal submission and subordination in dog–dog interactions [46]. Tail wagging is also used as an appeasement or affiliative signal in dog–human interactions [52,53]. One study found that during food denial situations, dogs wagged their tails more when a human was present versus not, suggesting that tail wagging may also function as a requesting signal [40]. Dogs frequently wag their tails when interacting with familiar and unfamiliar humans, but wag the most when their owners are present [34,54–57]. Dogs also wag their tails in response to non-social stimuli, such as food [23,27], fans [49] and plastic bags [35,49], with tail wagging in these situations thought to indicate positive emotions [23,27] and/or high arousal [23], but not fear [49] or stress [35].

### (d) Evolution

Tails are common across vertebrates and originally evolved for locomotion, with many animals also using tails for balance and swatting pests [51,58]. In canids, tails are no longer primarily used for locomotion [58], but rather for ritualized communication [51]. While dog tail wagging can vary by individual [59,60], sex [3,32,37,55] and breed [40,60,61], dogs wag their tails more frequently and in more contexts than any other canid [51]. Differences in dog and wolf tail wagging behaviour appear as early as three weeks of age, even when pups of both species have been raised in the same way [45]. In the next section, we investigate how and why this propensity for tail wagging evolved in dogs, focusing on a strong candidate trigger: the domestication process.

## 3. Effects of domestication on tail wagging behaviour

Domestication is defined as an evolutionary process arising from an ecological interaction: one species actively manages the survival and reproduction of another, which ensures resources and services to the former [62,63]. Domestication is a long process that ultimately leads to a range of physiological, morphological and behavioural changes in the domesticated species [64].

Dog domestication probably began during the Upper Palaeolithic period (approx. 35 000 BP) [65,66]. In domesticated dogs, and some other mammals, changes associated with domestication include: fur depigmentation [64,67], reduced facial skeleton and teeth size [67,68], changes in overall body size and proportions [69,70], the emergence of physical attributes like floppy ears and curled tails [67], reduced brain size [66,71], reduced aggression, increased docility and variation in hormone levels resulting in behavioural changes, such as a reduced response to stress [67,69,71,72]. In addition, comparative studies between wolves and dogs have shown that the domestication process shaped dogs' cognition and sociability in both dog–dog [73] and dog–human interactions [73–75]. Interestingly, dogs show a sophisticated ability to communicate and cooperate with humans: for example, in experimental tasks, they efficiently perceive and respond to human communicative cues, such as pointing and gaze [75–77].

Several hypotheses have tried to explain how these changes arose, outlining selective pressures that might have acted during domestication [78]. Desirable features in domesticated species are primarily the result of genetic selection by humans or an adaptation to a human-dominated environment. However, whether these traits emerged as a by-product of selection for other traits or were directly selected for is still unclear [66,67,79].

According to the ‘domestication syndrome’ hypothesis, domestication can lead to the emergence of genetically linked but unexpected traits, which are by-products of a more targeted trait selection [64,67] (but see [80] for a recent challenge). Changes in tail wagging behaviour could thus have arisen as a by-product of selection for another trait, such as tameness or friendliness toward humans. This aligns with results from a long-term experiment that tried to replicate the mammalian domestication process and track changes in behaviour, genetics and development in real-time. Silver foxes (*Vulpes vulpes*) were bred over 40 generations and directly selected for tameability and docility [81]. The resulting population of foxes exhibited behavioural, physiological and morphological traits similar to those observed in dogs (described above) [82,83]. Although tail wagging behaviour was not directly selected for, tamed foxes showed dog-like tail wagging behaviour and had more curled tails [64,81]. Based on this, we hypothesize that the domestication process may have led to changes at the behavioural and anatomical level that altered tail wagging behaviour in dogs, such that dogs wag more often and in more contexts than non-domesticated canids. This could have been due to a genetic link between the selection for tameness and tail anatomy. For instance, initial selections for docility may have triggered alterations of the neural crest cells during development, with repercussions on various phenotypic traits, including tail anatomy [72,84,85]. In line with other domestication hypotheses, such as the ‘deferential behaviour’ hypothesis [78] and the ‘emotional reactivity’ hypothesis [86], we suggest that, independently from the possible specific behavioural trait targeted during the domestication process, the altered tail wagging behaviour seen in dogs could have arisen as a direct expression of docility/friendliness.

Alternatively, tail wagging behaviour may have been one target of the domestication process, with humans (un)consciously selecting for dogs who wagged their tails more often, and potentially more rhythmically. We call this the ‘domesticated rhythmic wagging’ hypothesis. Tail wagging is a stereotyped, cyclical and rhythmic behaviour [87,88]. Extensive multidisciplinary evidence shows that humans have remarkable abilities to perceive and produce rhythmic sequences, particularly isochronous patterns where events are evenly spaced in time [89–96]. How this behavioural trait appeared in humans is still not clear, but cognitive neuroscience shows that human brains prefer rhythmic stimuli, which trigger pleasurable responses and engage brain networks that are part of the reward system [97,98]. This propensity for isochronous rhythms could have driven human selection for the conspicuous rhythmic wagging of the tail in dogs, and could explain why dogs exhibit it so often in human–dog interactions.

Under both hypotheses, selection on tail wagging behaviour may not have been uniform across breeds; for example, hunting-type dogs wag their tails more than shepherd-type dogs, and have also experienced different selective pressures throughout domestication [40,65]. Was tail wagging behaviour a by-product of selection for other traits (i.e. the ‘domestication syndrome’ hypothesis) or was it directly selected by humans because of its rhythmic properties (i.e. the ‘domesticated rhythmic wagging’ hypothesis)? Answering this question requires dedicated experiments that not only better quantify tail wagging in general but also explicitly consider how the behaviour is controlled.

## 4. Recommendations and future directions

Dog tail wagging may in fact be one of the most visible, prevalent animal behaviours in the world, but it has never been analysed in a systematic way. Most studies have measured when, for how long or at what rate tail wagging (broadly defined) occurs. These types of analyses are useful but limited in scope, and make teasing apart different evolutionary hypotheses challenging. Indeed, our brief review emphasizes that tail wagging is a multidimensional trait that can differ according to various parameters, including tail carriage, wag direction, wag speed, wag (a)symmetry and wag amplitude [59]. It can also vary depending on the portion of the tail being considered (i.e. base, central portion or tip). In theory, each tail movement parameter could be under different levels of neural control, have different functions and/or convey different information.

To better characterize the behaviour as a whole and distinguish between types of tail wagging, our first suggestion is to perform precise behavioural analyses of high-quality tail wagging videos [20], in concert with newly developed automated tracking tools specific to this oscillatory behaviour [99]. By simultaneously quantifying the parameters mentioned above, we can start to determine whether and how they relate to each other. Such analyses should be performed with dogs exposed to different stimuli (e.g. social versus non-social; of positive, neutral or negative valence) and accompanied by physiological measurements (e.g. heart rate, heart rate variability, cortisol, oxytocin, serotonin, testosterone); this will clarify how context and physiology impact different tail wagging parameters.

Second, we suggest that combining techniques of behaviour, computer vision and physiology analysis with neuroscience can help disentangle between tail movements under control (thus, under possible selection) from those resulting from mere mechanical effects (e.g. the tip of the tail might move as a consequence of more cranial portions of the tail being actively moved). Dogs are one of few animals, apart from humans, for which both non-invasive electrophysiology (e.g. EEG) and neuroimaging (e.g. fMRI) have been developed [100–103]. Neuroimaging techniques will help pinpoint which brain areas and networks are involved in tail wagging perception and production. Electrophysiology will support mapping the temporal dynamics of the putative involvement of different areas in the dog's cerebral cortex. Better understanding of tail wagging parameters and control will allow us to answer many outstanding questions, including investigating different evolutionary hypotheses for dog tail wagging. Table 1 lists outstanding questions and suggested methods to answer them.

**Table 1.** Outstanding questions on dog tail wagging behaviour and related suggestions for future studies.

Tinbergen domain	outstanding questions	methods
mechanism	physiology: are different tail wagging parameters associated with different arousal levels and/or physiological mechanisms?	—systematically quantify dog tail wagging parameters (see ‘Recommendations and future directions' section for details) while collecting real-time physiological (e.g. heart rate, cortisol, oxytocin) and behavioural (e.g. pacing, yawning) measures of arousal [22] in different contexts (e.g. affiliative, aggressive)
	biomechanics: is the entire tail under neurological control, or just a portion of it (e.g. base, tip)? Which brain circuits control tail wagging? Is any parameter of tail wagging under voluntary control or potentially learned?	—monitor dog tail activity while simultaneously conducting non-invasive electrophysiology (e.g. EEG) and neuroimaging (e.g. fMRI) [100–103].
	genetics: how much inter-individual phenotypic variation is there in tail wagging? How much of that variation can be explained by genetics?	—use whole-genome sequencing data and comparative genomics to investigate whether tail wagging is a genetically linked trait; a logical starting point would be to build on genomic research that has already been conducted on dog tail length [104] and shape [105].
ontogeny	development: does tail wagging behaviour in the same individual change from puppyhood to adulthood? Is any age-related variability in tail wagging linked to cognitive development and/or the production of other communicative signals?	—systematically quantify dog tail wagging parameters in the same individuals throughout development while also tracking changes in cognition and non-tail-based communication [106,107].
function	context: how do dog tail movements differ in social versus non-social contexts?	—systematically quantify dog tail wagging parameters when dogs are exposed to social and non-social stimuli and situations (e.g. [57]).
	audience: are there systematic differences in tail wagging behaviour when dogs are interacting with humans versus with conspecifics? How do receivers perceive different tail wagging parameters?	—systematically quantify dog tail wagging parameters in both intra- and inter-specific contexts (for an example of how differences in dog–dog and dog–human interactions can be investigated, see [108]) and investigate the effect on the receiver (human or conspecific, e.g. [22]).
evolution	phylogeny: which features of dog tail wagging are shared with other members of the class (mammals), suborder (caniforms) and family (canids)? Which features are more recent phylogenetic innovations?	—systematically quantify tail wagging parameters in a diversity of species and compare the resulting phylogeny of behavioural similarity (in terms of tail wagging) with a phylogeny of genetic relatedness (e.g. [109]).
	selection: is increased tail wagging in dogs a by-product of selection for other traits or was it directly selected? Is tail wagging correlated with other domestication traits (e.g. docility)?	—compare dog tail wagging with tail wagging in other domesticated mammals (e.g. pigs or cats) to shed light on whether tail wagging results from domestication in general (i.e. a general selection for tameness) or is specific to dog domestication. —quantify correlations between temperament traits such as docility, friendliness or deferentiality (as measured through temperament test batteries, e.g. [110]) and tail wagging parameters.
	perception: do humans and dogs have a preference for more rhythmically regular tail movements?	—test the perception of tail wagging parameters in humans and dogs (and ideally in non-human primates and other canids as well) through neuroimaging and physiological studies (e.g. expose both humans and dogs to tail wagging dogs and measure attention parameters such as eye fixations) [111–114]. —human studies should be conducted on diverse participants, given that age, cultural upbringing, and personal experience with dogs could all be confounding factors. —testing human populations living in close proximity to free-ranging dogs would be particularly interesting from an evolutionary perspective, since an unconscious positive selection for dogs wagging their tails, mediated by a potential preference for their rhythmicity, could still be taking place.

## 5. Conclusion

Dog tail wagging is a conspicuous yet scientifically elusive behaviour. Its uniqueness, complexity and ubiquity have the potential to be associated with multiple functions, but its mechanisms and ontogeny are still poorly understood. These knowledge gaps prevent us from fully understanding the evolutionary history of modern tail wagging behaviour and what role humans played in the process. A more systematic and thorough investigation of tail wagging will not only better map this iconic dog behavioural display, but also provide indirect evidence into the evolution of human traits, such as the perception and production of rhythmic stimuli.

## Data Availability

This article has no additional data.
